# Differing radiation exposure in scrub technicians and rotating staff in Cardiac catheterization laboratory: occupation matters

**DOI:** 10.1186/s43044-024-00448-8

**Published:** 2024-02-09

**Authors:** Muhammad Nasir Rahman, Maleeha Javed, Ghufran Adnan, Maria Khan, Zeenat Nizar, Izat Shah

**Affiliations:** 1 1Department of Medicine, Section of Cardiology, Aga Khan University Hospital, National Stadium Road, Karachi, Pakistan; 2 Aga Khan University Hospital, National Stadium road, Karachi, Sindh Pakistan; 3 Wazirabad Institute of Cardiology, Wazirabad, Pakistan; 4 Section of Cardiology, Department of Medicine, Aga Khan University Hospital, National Stadium Road, Karachi, Pakistan

**Keywords:** Radiation exposure, Non-physician staff, Occupational role, Coronary angiography

## Abstract

**Background:**

Radiation exposure is a significant hazard associated with invasive Cardiology, with most studies based on primary operator exposure. This prospective, observational study aimed to find out over lead radiation exposure as effective dose acquired by non-physician staff comprising scrub technicians and rotating staff in the cath laboratory. Effective dose (ED) measured per procedure via Raysafe i2®dosimeter badges worn by both rotating staff and scrub technicians over lead aprons along with dose area product (DAP), fluoroscopy time (FT) and procedure time (PT) in minutes was collected prospectively over forty-six invasive Cardiology procedures.

**Results:**

This study shows that rotating staff acquire higher ED in comparison with their scrub technician colleagues in diagnostic, interventional and electrophysiology cases. However, a statistically significant difference in radiation exposure of both staff groups was demonstrated in diagnostic and interventional Cardiology procedures, with *p* values of 0.04 and 0.01, respectively.

**Conclusions:**

These findings highlight occupational role and mobility around fluoroscopic sources as major factors in radiation exposure, which should be addressed within current radiation protection practices.

## Background

The advent of interventional Cardiology over the past few decades has shed light on the attendant risk of long term, repetitive radiation exposure, as an occupational hazard for physicians and staff working in the Cardiac catheterization laboratory [1, 2].

The recognition of radiation exposure as a significant health risk imposing both tissue reactions and stochastic effects [3] has brought in place protective measures such as lead aprons, neck collars and lead shields [4]. Apart from aforementioned protective lead equipment, it is now an essential practice to ensure measurement of radiation exposure of cath laboratory personnel. Effective dose, as a measure of individual absorbed dose serves to provide feedback as to occupational exposure and related risks, especially with regard to the International Commission on Radiological Protection (ICRP) limits of 100 msV whole-body effective dose over five years and 50 msV in any single year [5].

Non-physician staff are a pivotal part of the procedural team, comprising of scrub technicians involved as secondary operators and as registered nurses assisting in patient care, monitoring and medication administration during invasive procedures.

There is a dearth of regional studies on frequent, procedure wise effective dose measurement using external personal dosimeter badges. This is an inconsistent method which lacks feedback of procedural characteristics, leading to increased exposure. There is also a lack of special focus on non-physician cath laboratory staff [6, 7], with current radiation protection measures largely based on increasing distance and shielding from fluoroscopy source [8]. This pilot study was carried out exclusively to study radiation exposure of non-physician cath laboratory personnel functioning as scrub technicians and rotating staff, using dosimeter badges worn over equipment to measure scatter radiation exposure of areas that remain unprotected by standard lead apron such as head, lens and upper limbs [9].

## Methods

Nine staff members participated in this study, provided with Raysafe i2® dosimeter badges for each procedure after informed consent. This group consisted of two female and seven male participants of which five members were registered nurses functioning as rotating staff, and four were scrub technicians. Average ages were between 25 and 55 years. Radiation data comprised ED, DAP, fluoroscopy times (FT) and procedure times (PT). This was obtained prospectively over four months from March 2021 to June 2021 for forty-six invasive adult Cardiology procedures which were mainly diagnostic coronary angiograms (CA), percutaneous coronary interventions (PCI), combined CA, PCI cases and electrophysiology (EP) procedures with right radial arterial access, subclavian venous and right femoral arterial access utilized. Primary operators variably comprised of two electrophysiologists, two noninvasive Cardiologists and four intervention Cardiologists along with seven fellows in training. Procedures took place in two fluoroscopy suites located in our cath laboratory. Artis Zees, Siemens® machines were utilized with DAP being measured via collimator, displayed digitally in external monitor, with cumulative FT being displayed as well as a sum of cine, and fluoroscopy acquisitions by operator. Procedure time was noted as time from obtaining arterial or venous access to removal of arterial/venous sheath at end of procedure. Effective dose, defined a dose quantity defined as the sum of the tissue-equivalent doses weighted by the ICRP organ (tissue) weighting factors**,** which takes into account the varying sensitivity of different organs and tissues to radiation.

### Dosimetry

Raysafe i2® system was used for the purpose of this study. This comprises of dosimeter badges with computer software providing real-time feedback during procedures. Two monitors, one employed as live display of radiation exposure as ED of every staff member during fluoroscopic acquisition, serve to implement immediate measures to reduce intra-procedural ED such as increasing distance from fluoroscopy source. The other monitor used as a log of ED acquired post-procedure (see Fig. 2). The monitor log had preprogrammed calibration properties; hence, no correction factor or equations were used to obtain ED measurements.

Raysafe® external dosimeters were worn at neck level above thyroid neck collar by all participants (see Fig. 2). The badges did not alarm; hence, there was no real-time feedback during procedures. At the end of every procedure, badges were duly logged into the Raysafe® digital feedback system, providing measured effective dose in micro Sieverts (µ Sv). These external dosimeter badges were only provided to rotating staff and scrub technicians in each procedure, with no concomitant ED measurements for primary operators obtained.

**Fig. 1:**
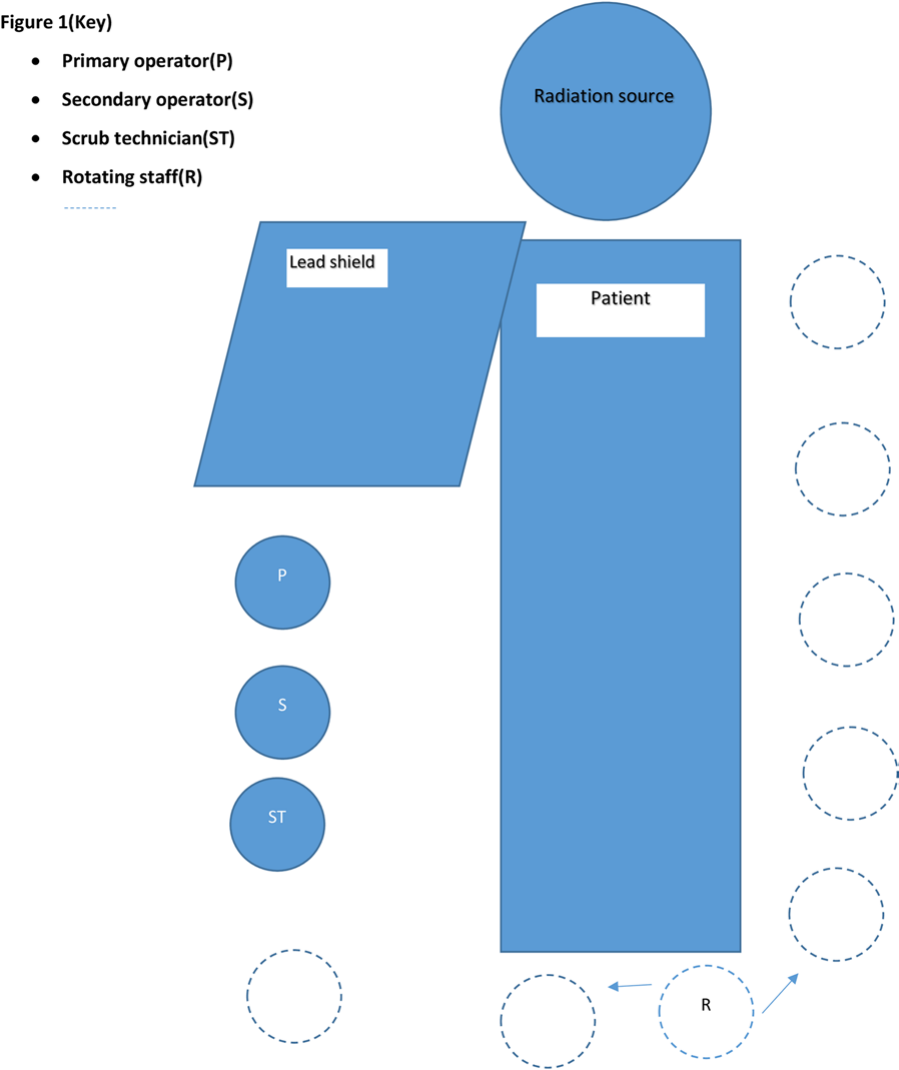
Diagrammatic representation of cath laboratory team, fluoroscopic source and lead shield

All staff wore 0.5-mm-thick lead personal protective equipment available as wrap around aprons extending below knee level, with appropriately sized thyroid collars. Two ceiling mounted and floor mounted lead shields were also employed, at right side of fluoroscopic source serving to protect operators and staff at right side of the patient. (See Fig. 1).

The procedural team comprised of two physician staff, usually faculty members or fellows in training along with residents at our institute, with non-physician staff members serving as scrub technicians functioning as secondary operators or assistants to secondary operators helping with device exchanges, manifold handling and dye injections. Physician staff and scrub technicians occupied static positions, with added protection from ceiling and floor mounted lead shields. (Fig. 1).

Rotating staff in our procedural team were circulatory nurses who had the primary responsibility of administering medications, adjusting monitor equipment and providing catheters and devices to primary operator as requested, a role which required mobility in cath laboratory, placed them at greater proximity to patient and fluoroscopic source, and without the protection of right-sided lead shield as compared to their scrub technician counterparts. (Fig. 1).

### Statistical analysis

We analyzed the data using statistical software STATA (version 14.2). Continuous variables were expressed as mean and standard deviation. Comparison was done by t test with two-sided *p* value utilizing less than 0.05 as significant was used with a confidence interval of 95% was used to compute the difference in radiation exposure after assessing normality. Calculations were done separately for each variable between both staff groups with significant *p* value less than or equal to 0.05.

## Results

Data were collected for forty-six invasive Cardiology procedures, comprising fourteen diagnostic coronary and graft angiograms (CA), thirteen CA and PCI, six complex PCI (percutaneous coronary intervention) cases entailing left main coronary interventions, bifurcation stenting, multi-vessel PCI and intervention for chronic total occlusion (CTO), four CA and FFR (fractional flow reserve) cases, two simple PCI cases and one ASD (atrial septal defect) closure case. There were six electrophysiology cases with three CRT (cardiac resynchronization therapy) device placements, two PPM (permanent pacemaker) insertion and one complex electrophysiology and ablation procedure.

Procedure time (PT), described as time in minutes measured from time to arterial or venous sheath insertion to its removal at end of procedure, was 69.28 per case ( SD: 46.70). (Table 1).

**Table 1:** Mean procedure time (PT), fluoroscopy time (FT) and dose area product (DAP) noted across 46 invasive Cardiology procedures

Procedures	Mean PT (min)	Mean FT (min)	Mean DAP (µgyn/m^2^)
N:46	69.28 (SD: 46.70)	19.53 (SD: 14.78)	9085.41 (SD: 8729.59411)

Fluoroscopy time for all 46 procedures was 898.70 min, mean 19.53 (SD: 14.78) (Table 1).

Total dose area product (DAP) was 417,928.96 uGycm2, with mean DAP measured as 9085.41 (SD: 8729.59) (Table 1).

Collectively, across all 46 procedures, markedly increased radiation exposure was observed as acquired by rotating staff, demonstrating mean ED.R as 21.04 µ Sv (SD: ± 39.64) and mean ED.S: 7.54 µ Sv (SD: ± 17.23), this almost threefold difference in radiation exposure was statistically significant with a *p* value of 0.03. (Table 2).

**Table 2:** Mean radiation exposure as in effective dose (ED) acquired by rotating staff (ED.R) and scrub technicians (ED.S)

All procedures	Mean ED.R: (µ Sv)	Mean E.D S: (µ Sv)	*p* value
(n:46)	21.04 µ Sv (SD: ± 39.64)	7.54 µ Sv (SD: ± 17.23)	**0.03**

The difference in ED reflecting greater radiation exposure of rotating staff (ED.R) was both numerically and statistically significant with a p value of **0.03**

Mean effective dose acquired by rotating staff (ED.R) in fourteen diagnostic cases was 3.85 µ Sv (SD: ± 4.72 µ Sv) as compared to that acquired by scrub technicians with mean ED.S: 1.14 µ Sv (SD: ± 0.53). This difference in mean effective dose acquired by both groups in diagnostic procedures was significant as par T test comparison, with *p* value of 0.04. (Table 3).

**Table 3:** Mean effective dose acquired by staff during diagnostic angiograms (DA), percutaneous coronary interventions (PCI) and electrophysiology procedures (EP)

Procedure type	Mean ED.R (µ Sv)	Mean ED.S (µ Sv)	*p* value
DA(n:14)	3.85 µ Sv (SD ± 4.72 µ Sv)	1.14 µ Sv (SD: ± 0.53)	**0.04**
Combined: (DA + PCI/FFR): (n:17)	22.55 µ Sv mean ED (SD: ± 17.52)	5.00 µ Sv (SD: ± 4.82)	**0.01**
PCI: (n:9)	18.76 µ Sv (SD: ± 23.71)	13.71 µ Sv (SD: ± 4.82)	0.56
EP (n:6)	65.33 µ Sv (SD: ± 92.71)	8.83 µ Sv (SD: ± 9.62)	0.16

These findings were consistent across the nine percutaneous coronary intervention procedures included in this study, with rotating staff acquiring 22.55 µ Sv mean ED ( SD: ± 17.52) as compared to scrub technicians who acquired a mean ED of 5.00 µ Sv (SD: ± 4.82), with a statistically significant difference in their radiation exposure with a *p* value of 0.01 obtained.. (Table 3).

Seventeen combined procedures entailing concomitant diagnostic and coronary interventions were part of this study, in which mean ED.R was 18.76 µ Sv (SD: ± 23.71), as compared to mean ED.S: 13.71 µ Sv (± 26.72). This difference in acquired radiation exposure was not significant, with *p* value of 0.56. (Table 3).

Six electrophysiology procedures demonstrated mean ED.R: 65.33 µ Sv (SD: ± 92.71) and mean ED.S: 8.83 µ Sv (SD: ± 9.62). Difference in acquired mean ED was not significant in this procedure sub group as well, with a *p* value of 0.16. (Table 3).

## Discussion

In this study, rotating staff had markedly higher radiation exposure, as compared to their scrub technician counterparts. The difference in acquired effective dose (ED.R and ED.S) was numerically higher among rotating staff across all procedures, with statistically significant differences seen in diagnostic angiograms and combined diagnostic and interventional Cardiology procedures (See Table 3) However, this was not the case in electrophysiology and combined diagnostic and coronary intervention procedures, whereby the difference in radiation exposure was not statistically significant.

This is a unique demonstration of occupational role as a significant factor affecting radiation exposure in non-physician staff in the cardiac catheterization laboratory, as opposed to most studies which focus on primary operator methods and procedural factors [10–12].

**Fig. 2:**
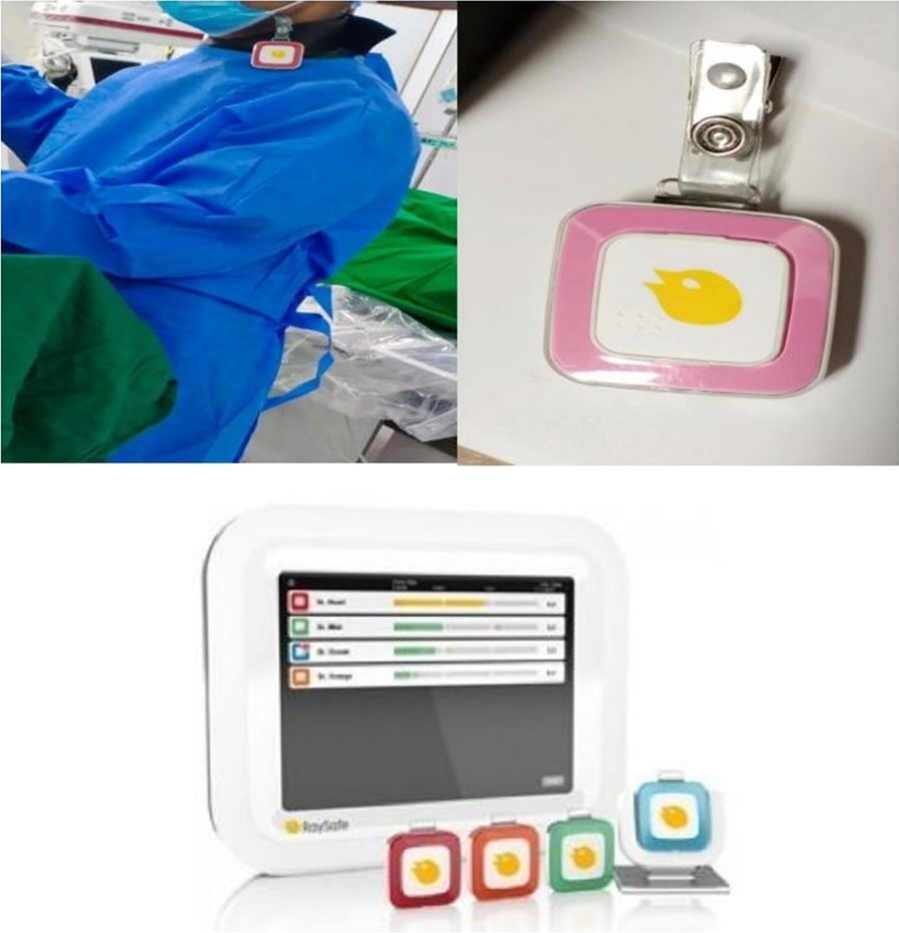
Clockwise from above right: Dosimeter badge worn by staff above lead apron, below: badge placed in cradle connected to monitor to obtain effective dose value

Rotating staff were exposed to greater amounts of radiation as they work in proximity to patients, administering medication, handing devices to operators, adjusting leads and monitors and often stand at the left side of the patient without any lead shield. (Fig. 2). Similar heightened radiation exposure has been reported in non-operator staff in the cath laboratory, such as anesthesiologists and echocardiographers [13], attributed to lack of left-sided lead shields.

On the contrary, scrub technicians work at a static position with additional protection from lead shields and assistants to primary operators prone to radiation exposure while performing hand injections, helping in device exchange. This effect of occupational role is demonstrated by highest ED.S acquired by scrub technicians in combined coronary diagnostic and intervention procedures. (See Table 3).

To the best of our knowledge, occupational role as a factor affecting individual radiation exposure in non-physician staff has only been highlighted by Madder RD et al., with increased radiation dose exposure of rotating staff related to their proximity to patients specifically in PCI and FFR cases [14]. In our study, greater radiation exposure of rotating staff extends to all procedure types, as shown by increased ED.R acquired in diagnostic, coronary interventions and electrophysiology procedures (Table 2.)

This very knowledge of radiation exposure related to occupational roles using real-time dosimetry may lead to interventions to reduce radiation exposure, as demonstrated by Murat et al. [15] whereby awareness of individual exposure led to a sixty percent decrease in subsequent exposure with optimized protection.

This was a small observational, single-center pilot study, with a limited number of cases, especially with regard to electrophysiology. Moreover, dosimeter badges were not widely available due to resource constraints.

The very idea of real-time dosimetry with individual radiation exposure measurement for each procedure is a novelty in our lower-middle-income country whereby standard cath laboratory practices exclude such measures. Further studies stemming from this preliminary study are much needed, to examine the impact of knowing individual real-time radiation exposure and modifying protective practices accordingly in our resource-limited setting. Also, more scrutiny of complex coronary interventions such as chronic total occlusion PCI, structural heart interventions and electrophysiology procedures is needed with regard to their individual impact on real-time radiation exposure.

## Conclusions

This new insight into radiation awareness calls for further protective measures with regard to rotating staff. Education regarding occupational roles and strategic positions should be provided to all cath laboratory personnel. Operator behavior modification of avoiding fluoroscopy use while rotating staff performs duties involving proximity to the patient is mandatory. Other essential measures should include accessory left-sided lead shields, mandatory protective lenses, head shielding equipment and shift-based duties for rotating staff during prolonged procedures with longer fluoroscopy times.

## Data Availability

The datasets used and/or analyzed during the current study are available from the corresponding author on reasonable request.

## Abbreviations

ED: Effective dose
ED.S: Effective dose acquired by scrub technicians
ED.R: Effective dose acquired by rotating staff
DA: Diagnostic angiography
PCI: Percutaneous coronary intervention
FFR: Fractional flow reserve
EPS: Electrophysiology procedures
